# The challenge of staying up-to-date in critical care

**DOI:** 10.62675/2965-2774.20260344

**Published:** 2026-01-28

**Authors:** Rob Mac Sweeney

**Affiliations:** 1 Critical Care Reviews Belfast United Kingdom Critical Care Reviews - Belfast, United Kingdom.

Non-implementation of new evidence is a longstanding issue in medicine. In critical care, the CHEST^(1)^ and 6S^(2)^ trials identified clear harm with the use of hydroxyethyl starch solutions. Yet, they continued to be widely used until regulatory approval was removed^(3)^ or modified.^(4)^ The PROSEVA trial^(5)^ identified a ~50% absolute mortality reduction from prone-position ventilation in patients with moderate-to-severe acute respiratory distress syndrome, yet despite this massive effect size, the uptake of this intervention remained low.^(6)^ While scepticism and implementation barriers play roles, a significant obstacle is simple unawareness. Clinicians cannot apply evidence they do not know exists.

The traditional approach to answering clinical questions at the bedside identifies only information that fits the narrow frame of a current patient problem. This strategy is reactive: it helps resolve an individual uncertainty, but can miss the vast majority of new, practice-changing evidence that never happens to be queried in real time. A more complex problem is systematically tracking the literature to maintain broad evidential awareness.

The scale of the medical literature is enormous and growing rapidly. As of March 2025, the PubMed database included over 39 million citations.^(7)^ During 2023, it added 1.57 million new papers, averaging 130,000 per month or 4,300 per day.^(8)^ The MEDLINE database, forming the central component of PubMed, includes over 5,200 journals^(8)^ across 40 languages. A MEDLINE search for critical care or intensive care medicine journals lists 156 journals (although over 20 are not relevant),^(9)^ and many more exist outside MEDLINE. Numerous general medical and specialty journals also publish critical care-related material, making the scope of publication of potentially relevant papers vast and spanning much of the entire medical literature. Publication pollution from predatory journals, numbered at 86 in critical care in 2019, compounds the signal-to-noise ratio.^(10)^

Journal publications remain the primary route for disseminating scientific findings, but are not the only route. Over the past decade, preprint servers have become common venues for sharing results prior to peer review, and many pivotal trial results or guidelines are first presented at major conferences, both within and beyond critical care. Commercial sponsors sometimes announce results by press release, further fragmenting discovery pathways.^(11)^

Access barriers also limit awareness. Paywalls for journal and conference content are common, and language remains a structural hurdle. While there are over 7,100 languages worldwide, most medical publications are in English, despite only 7.3% of the global population speaking English as a first language and 12% as a second language.^(12)^ A third major impediment to staying up-to-date is the time required, not just to read and understand the latest evidence, but to simply find the most relevant material in a constant stream of information.

Multiple digital resources can help stay up to date. New information releases, via primary media such as journals, conferences, press releases, and preprint servers, can be amplified by secondary resources, such as microblogging sites, medical websites, and blogs. Push alerts, enabling free emails to be sent automatically, allow subscribers to stay current with an emailed table of contents and new article alerts for journals and preprint servers. Really Simple Syndication (RSS) feeds, used by journals, websites, and other alert systems, automatically update with new content and are captured by an RSS aggregator, so all feeds come to a single location. Independent blogs and podcasts can disseminate material, as can those from societies and journals. Due to the burden of creating this content, this is a limited way to stay up to date in general, but it is excellent for understanding specific material in depth. Automated searches via PubMed, Google Scholar, and the Cochrane database can help track specific topics, but subsequent searches must be checked by hand. EvidenceAlerts from McMaster University in Canada tracks 121 journals and sends alerts when new publications match a pre-specified topic of interest. Read by QxMD is an update platform for tracking the literature, offering both free individual and paid institutional subscriptions. Up-to-Date and DynaMed are widely used proprietary clinical decision support tools and provide contemporary overviews of conditions, investigations, and therapies. Both primarily focus on providing on-demand clinical consultation. DynaMed does have a literature surveillance feature through its update alerts, but this is not the platform's central aim.

The digital age has evolved into the Artificial Intelligence (AI) age, with a plethora of apps offered to manage the research landscape. Just as with large language models in general, these still require human expert oversight, as the risk of inaccuracies and hallucinations persists. OpenEvidence^(13)^ has successfully launched to verified clinicians in the United States, with a more limited version offered internationally. Competitors, such as Doximity,^(14)^ which are moving into medical AI and the clinical reference space, will provide alternative options. These AI tools will likely supersede Up-to-Date and DynaMed in their current form for bedside clinical decision support. Whilst these provide contemporary, evidence-based answers to clinical questions in real time, their ability to manage large-scale surveillance of the literature is presently less clear.

Critical Care Reviews^(15)^ (CCR) pairs daily Journal Watch surveillance with a weekly newsletter, as well as Hot Trials & Guidelines, an annual book that summarises and critiques pivotal trials, and meetings in Belfast and Melbourne that highlight new evidence. The Foundational Trials Collection curates the key studies underpinning contemporary practice. With free access to almost all resources (and newsletter access waived for clinicians in low and lower-middle-income countries), CCR addresses cost barriers while compressing time to awareness.

Staying up to date in critical care is vital to delivering optimal care; yet the volume, dispersion, access, language, and time barriers make this problematic. Artificial Intelligence, combined with human curation, embedded in workflows and backed by a desire to stay current, is the most credible path forward as the ecosystem continues to evolve (Figure 1).

**Figure 1:**
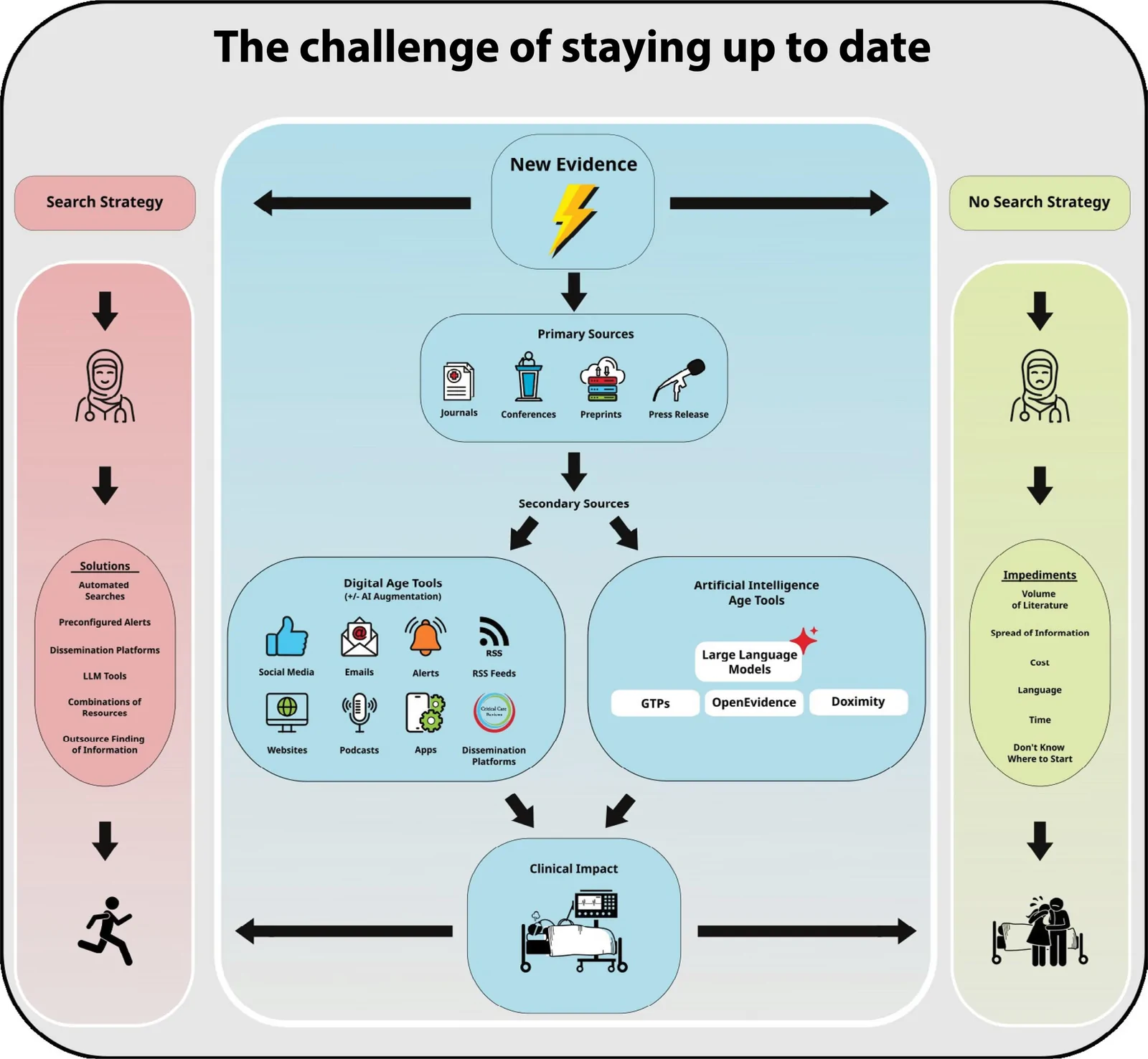
The challenge of staying up to date in critical care.

LLM - large language model; RSS - Really Simple Syndication; GTP - generative pre-trained transformer

## Data Availability

The contents underlying the research text are included in the manuscript.

## References

[1] Myburgh JA, Finfer S, Bellomo R, Billot L, Cass A, Gattas D (2012). Hydroxyethyl starch or saline for fluid resuscitation in intensive care. N Engl J Med.

[2] Perner A, Haase N, Guttormsen AB, Tenhunen J, Klemenzson G, Åneman A (2012). 6S Trial Group; Scandinavian Critical Care Trials Group. Hydroxyethyl starch 130/0.42 versus Ringer's acetate in severe sepsis. N Engl J Med.

[3] European Medicines Agency (EMA) (2018). Hydroxyethyl-starch solutions for infusion to be suspended – CMDh endorses PRAC recommendation.

[4] FDA. Food & Drug Administration (2021). Labeling Changes on mortality, kidney injury, and excess bleeding with hydroxyethyl starch products.

[5] Guérin C, Reignier J, Richard JC, Beuret P, Gacouin A, Boulain T (2013). PROSEVA Study Group. Prone positioning in severe acute respiratory distress syndrome. N Engl J Med.

[6] Bellani G, Laffey JG, Pham T, Fan E, Brochard L, Esteban A (2016). LUNG SAFE Investigators; ESICM Trials Group. Epidemiology, patterns of care, and mortality for patients with acute respiratory distress syndrome in intensive care units in 50 countries. JAMA.

[7] National Library of Medicine (NIH) PubMed. About PubMed.

[8] National Library of Medicine (NIH) PubMed. MEDLINE PubMed Production Statistics.

[9] National Library of Medicine (NIH) NLM Catalog.

[10] Cortegiani A, Sanfilippo F, Tramarin J, Giarratano A (2019). Predatory open-access publishing in critical care medicine. J Crit Care.

[11] Spectral Medical INC (2025). Spectral Medical and Vantive Announce Topline Results from Spectral's TIGRIS Trial Evaluating PMX Hemoadsorption Therapy for Endotoxic Septic Shock.

[12] Bahji A, Acion L, Laslett AM, Adinoff B (2023). Exclusion of the non-English-speaking world from the scientific literature: recommendations for change for addiction journals and publishers. Nordisk Alkohol Nark.

[13] OpenEvidence About.

[14] Doximity (2025). News details. Doximity Acquires Pathway, a Leader in AI Clinical Reference.

[15] Mac Sweeney R (2025). Critical Care Reviews.

